# Opsoclonus-myoclonus syndrome in the course of teratoma: a case report

**DOI:** 10.3389/fmed.2024.1519408

**Published:** 2025-01-09

**Authors:** Mateusz Szczupak, Jacek Kobak, Anna Wiśniewska, Justyna Kosydar-Bochenek, Arkadiusz Jamro, Sabina Krupa-Nurcek

**Affiliations:** ^1^Department of Anesthesiology and Intensive Care, Copernicus Hospital, Gdańsk, Poland; ^2^Department of Otolaryngology, Faculty of Medicine, Medical University of Gdańsk, Gdańsk, Poland; ^3^Department of Neurology, Copernicus Hospital, Gdańsk, Poland; ^4^Institute of Health Sciences, College of Medical Sciences of the University of Rzeszow, Rzeszów, Poland; ^5^Student at Institute of Medical Sciences, Medical College of Rzeszów University, Rzeszów, Poland; ^6^Department of Surgery, Institute of Medical Sciences, Medical College of Rzeszów University, Rzeszów, Poland

**Keywords:** opsoclonus myoclonus, case study, gynecology, patient, teratoma

## Abstract

Opsoclonus-myoclonus syndrome (OMS) is a rare neurological inflammatory disease of paraneoplastic, parainfectious or idiopathic origin. It is manifested by the occurrence of opsoclonus, myoclonus, ataxia, as well as behavioral and sleep disorders. The incidence is estimated at 1/5,000,000 people. This syndrome is usually immune-mediated and may be the first manifestation of cancer as a paraneoplastic syndrome, most often occurring in the course of breast, ovarian or lung cancer. Here we show a case of a 20-year-old woman with symptomatic opsoclonus-myoclonus syndrome in the course of teratoma. A brief review of the literature was conducted to determine the diagnostic route and treatment of this rare condition. As a result, it has been shown that the only method of treatment for OMS syndrome is the removal of the neoplastic lesion.

## Highlights

- In the available literature, no other way of treating OMS syndrome has been found other than surgical treatment of tumor removal.
- Untreated OMS can cause sleep and behavioral disorders, which in turn can contribute to the development of complications from the nervous system and the need for treatment in a psychiatric clinic.

## Introduction

Opsoclonus-myoclonus syndrome (OMS) is a rare set of disease symptoms, usually immune-mediated, whose symptomatology consists of: opsoclonus (rapid, multidirectional, combined eye movements), myoclonus (sudden jerking movements involving the axial and limb muscles), ataxia (limb and axial of varying severity), sleep disturbances, irritability and other behavioral changes (1, 2). This syndrome was first described in the literature by the Austrian pediatric neurologist and neurobiologist Marcel Kinsobourn in 1962. In his work entitled “Myoclonic encephalopathy of infants,” he presented six cases of children aged 6–20 months who developed myoclonus generating ataxia and opsocloni (3). According to the literature, the term opsocloni was first introduced into medical nomenclature in 1913 by the Polish neurologist Professor Kazimierz Orzechowski (4). In 1927, the researcher linked opsocloni to myoclonus (1, 5). In the same year, American scientists Harvey Cushing and Simeon Wolbach described the relationship between opsocloni and myocloni and fetal sympathetic neuroma in children (6).

The aim of this publication was to present the case of a 20-year-old woman who was admitted to the neurology department due to rapid, multidirectional and combined eye movement accompanied by myoclonus and irritability. The symptoms appeared suddenly, and the diagnostic procedure during the stay in the hospital emergency department did not allow to determine their cause. This prompted the team of doctors caring for the patient to be admitted and further diagnosed as part of the neurology department of the Copernicus Hospital. The authors of this manuscript have reviewed the literature to determine the management and treatment of a patient with this syndrome.

## Materials and methods

In order to write this manuscript, the articles available in the PubMed, Google Scholar and Mendeley search engines were reviewed. Keywords such as opsoclonus-myoclonus syndrome, teratoma, ataxia, and multidirectional nystagmus were used. Out of 45 review articles and articles presenting OMS cases found and analyzed, those were selected which, in the opinion of the authors, referred to the subject of this manuscript and constituted a valuable source of information. Those that duplicated the pathophysiology of opsoclonus-myoclonus syndrome or referred to diseases that were not directly related to the topic were excluded from the discussion. The article cites 28 publications and scientific reports. The case of a 20-year-old female patient who was hospitalized due to symptoms in the Department of Neurology of the Copernicus Hospital, Nicolaus Copernicus Hospital in Gdańsk was also presented.

### Pathophysiology and symptomatology of opsoclonus-myoclonus syndrome

In adults, after excluding structural causes, the most common etiology (60% of cases) of opsoclonus-myoclonus syndrome (OMS) are neoplastic diseases (lung cancer, breast cancer, ovarian teratoma, kidney cell cancers or pancreatic malignant tumors) (7).

The etiopathogenesis of opsoclonus in OMS has still not been clearly understood (6). There are two hypotheses for the development of this syndrome. The first one is associated with the development of opsoclonus with dysinhibition (disinhibition) of the apical nucleus of the cerebellum. Under normal conditions, the nucleus of the cerebellar apex is inhibited by the dorsal part of the cerebellar vermis – in the case of disorders of the inhibition process, it is disinhibited. This theory is confirmed by functional neuroimaging studies. Single photon emission-computed tomography (SPECT) showed dysfunction of the dorsal part of the cerebellar verm, and functional magnetic resonance imaging (fMR) showed excessive bilateral activation of the cerebellar vertex nuclei (1, 8–10).

The second concept of OMS development is associated with immunological determinants and has a paraneoplastic or idiopathic basis. Paraneoplastic etiology occurs at the age of 60–70 years and is usually associated with small cell lung cancer, breast adenoma or ovarian cancer (7, 10–12). In some patients, antibodies against nerve cell surface antigens and neurofilament antigens can be identified (13, 14). As a result of the humoral response, oncogenic antibodies are produced – anti-Ri, -Hu, -Yo, −Ma1, −Ma2, -Ta, -CRMP-5, -CV2, antiamiphysin and neurofilaments. Their presence confirms the paraneoplastic basis of the disease, although the absence does not exclude this cause (1, 2, 7, 10, 11, 15, 16). In the majority of patients with paraneoplastic OMS, the presence of onconeuronal antibodies is not found (1, 11). The exception to this rule are women with OMS and breast or ovarian cancer, who have anti-Ri antibodies directed against the Nova protein, which regulates the function of other proteins involved in synaptic transport in the central nervous system (12, 17).

The idiopathic form of OMS usually affects people aged 30–40 years, and its occurrence is rarely preceded by infection or vaccination (1, 7, 18–20). The humoral immune response is directed against synaptic autoantigens or surface nerve cells, which explains the neuronal dysphyction present in idiopathic OMS (12). This theory is confirmed by Bataller et al., who identified postsynaptic autoantigens such as the bound protein complex in OMS patients. NMDA receptor (16). Similar conclusions were reached by Blaes et al., who discovered antibodies in patients with OMS that bind to the surface of neurons of the granular layer of the cerebellar cortex (21).

Opsoclonus-myoclonus syndrome is sometimes a manifestation of periinfectious encephatomyelitis of bacterial etiology (*Borrelia burgdorferi*, *Mycoplasma pneumoniae*) or viral etiology (enteroviruses, Ebstein-Barr virus, cytomegalovirus, West Nile, HIV) (7, 11, 22–25).

In the literature, there are also reports referring to the development of opsoclonus-myoclonus syndrome in the course of diabetic coma, celiac disease, pregnancy, allogeneic bone marrow transplantation, poisoning, drugs such as lithium or amitriptyline, psychoactive substances such as cocaine or toxic compounds such as strychnine or thallium. Sometimes OMS can be a portal effect of craniocerebral trauma, vascular diseases, demyelization diseases or cancers of the central nervous system (10, 11, 22, 23, 26).

In almost 30% of cases, the development of opsoclonus-myoclonus syndrome in adults is preceded by the appearance of parainfluenza symptoms. The most common of these include: non-systemic dizziness, balance disorders, nausea and/or vomiting or visual disturbances (6, 11).

In most cases, both opsoclonus and myoclonus are observed from the beginning of the syndrome. Opsocloni can be observed during eye fixation, smooth tracking or convergent eye movementscg. They are also present when the eyelids are closed and during sleep. They require differentiation from “eye flutter” and nystagmus (6). Myoclonus, on the other hand, are sudden arrhythmic, multifocal contractions of the muscles of the limbs, less often of the head, neck or trunk. Myoclonus can occur in certain positions – positional myoclonus or be provoked by movement – kinetic myoclonus. They are most often intensified under the influence of external stimuli and emotional reactions (1, 2, 6, 11). The development of trunk ataxia in the course of OMS, which is a symptom of cerebellar syndrome, is sometimes associated with the development of limb ataxia and dysartia. Both ataxia and myoclonus in about half of patients lead to disability within a month as a result of gait disorders, falls, difficulties in standing and sometimes also sitting (1, 2, 6, 11). Behavioral disorders as a result of encephalopathy associated with opsoclonus-myoclonus syndrome may manifest as confusion, attention deficit, emotional lability or mood disorders (1, 6, 11).

## Case presentation

A 20-year-old woman was admitted to the Department of Neurology at the Copernicus Hospital due to increasing balance disorders, inability to walk, dizziness with a feeling of spinning of the environment, a sensation of “jumping” image and difficulty fixing her eyes. In addition, nausea and vomiting occurred. The appearance of symptoms was preceded by severe headache located in the frontal area, with their increasing intensity 3 days before hospitalization. The other neurological symptoms gradually worsened, leading to significant balance difficulties and gait disorders, with maximum intensity on the day before hospital admission. Medical history: patient with polycystic ovary syndrome, insulin resistance, obesity with Body Mass Index 42.4 (BMI 42.4), hypothyroidism, after stabilization surgery and Th4-L1 spondylodesis due to idiopathic juvenile scoliosis in 2016, without a history of previous neurological or psychiatric diseases. Neurological examination at admission to the ward was dominated by severe oculomotor disorders in the form of chaotic, irregular eye movements, intensifying when trying to fix the gaze. In addition, features of the cerebellar syndrome were observed in the form of upper limb dysmetria, trunk ataxia, unsteadiness in the Romberg test, and gait on a wide base. Other symptoms included resting tremor of the limbs and trunk, involuntary movements of the limbs and trunk in the form of myoclonus, and right-sided pyramidal symptoms. To determine the cause of these symptoms, a number of diagnostic tests were carried out. Magnetic resonance imaging of the head described a non-specific small high-signal focus in the cortex of the lower part of the left cerebellar hemisphere. The electroencephalographic (EEG) recording was abnormal – the presence of single sharp wave discharges was found in the frontotemporal and temporal leads, mainly the left ones. In addition, numerous artifacts from eye movement were noted in the frontal leads. The results of laboratory tests were within the reference levels of. No abnormalities were observed in the general examination of cerebrospinal fluid (Table 1). The level of anti-IgM and anti-IgG antibodies to *Borrelia burgdorferi*i in serum and cerebrospinal fluid was negative. Similarly negative was the PCR panel for 14 neuronal infection pathogens (cytomegalovirus, enterovirus, herpes simplex virus type 1, herpes simplex virus type 2, human herpesvirus type 6, human parechovirus, varicella zoster and herpes zoster virus, *Escherichia coli* K1, Hemophilus influenzae, *Listeria monocytogenes*, *Neisseria meningitidis*, Streptococcus agalactiaem *Streptococcus pneumoniae*, *Cryptococcus neoformans*/GATTII) and the level of antibodies for atypical encephalitis.

**Table 1:** Result of the general cerebrospinal fluid examination.

Tested parameters	Result	Reference range
Protein	27,8 mg/dL	15–45
Glucose	55 mg/dL	40–70
Chlorides	124 mmoL/L	118–132
Cytosis	6 komórek/μl	0–5
Albumin	225 mg/L	100–300
IgG	33,5 mg/L	10–30
MN% - percentage of cells with a monolobed nucleus	100%	–
PMN% - percentage of cells with a multilobed nucleus	0%	–
Color before centrifugation	Waterlight	–
Color after centrifugation	Waterlight	–
Transparency before centrifugation	Transparent	–
Clarity after centrifugation	Transparent	–
Erythrocytes in cerebrospinal fluid	Absent	–

**Figure 1:**
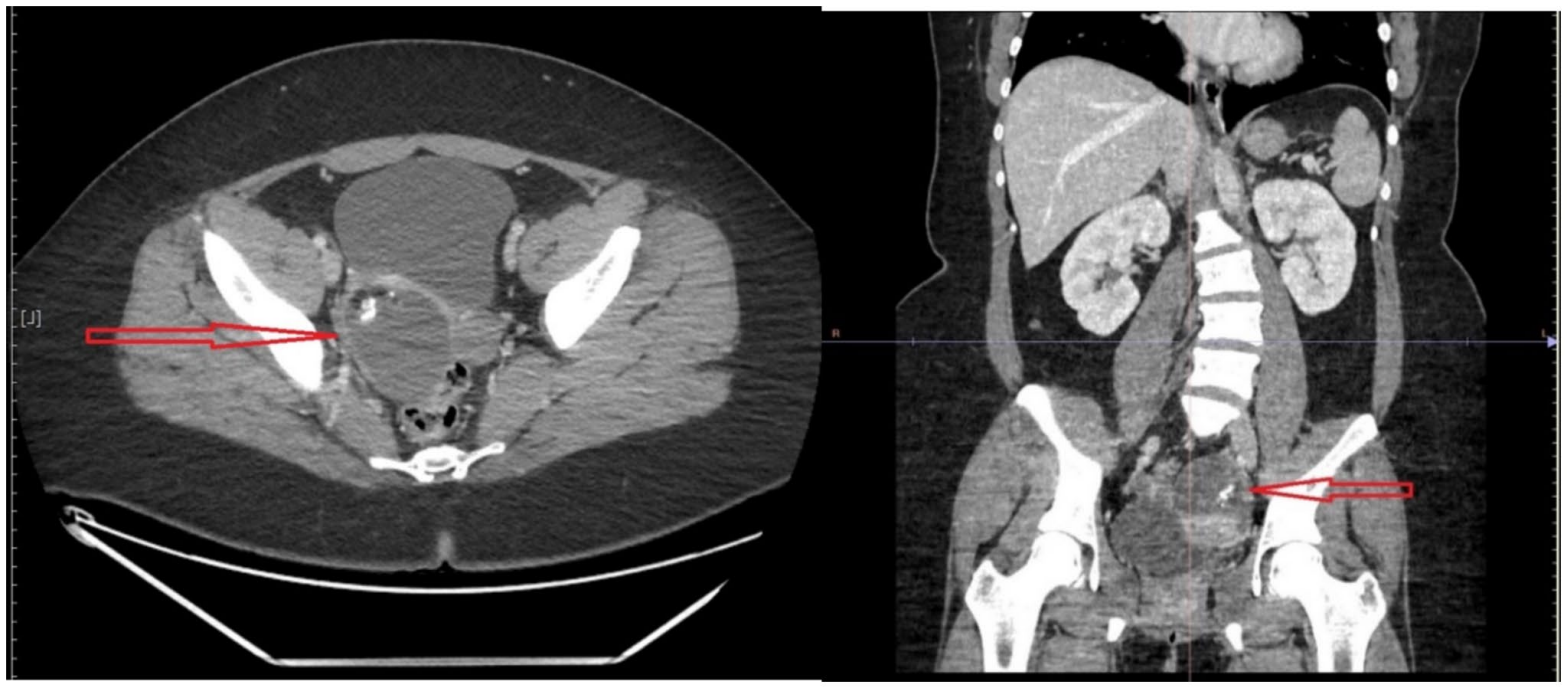
Computed tomography of the chest, abdomen and small pelvis with marked pathological mass of the left ovary.

In the course of further diagnostics, computed tomography (CT) of the chest, abdominal cavity and small pelvis was performed, in which a well-demarcated mass in the left ovary measuring 56x77x66 mm suggestive of a teratoma was described from the pathological changes (Figure 1). Transvaginal ultrasonography was performed, which confirmed the presence of a lesion with an ultrasound appearance typical of a teratoma.

Due to the overall clinical picture suggesting the development of opsoclonus-myoclonus syndrome in the course of teratoma, the team caring for the patient additionally performed immunoblot tests for the presence of onconeuronal and antineuronal antibodies and the ROMA test – negative results were obtained. During hospitalization, after collecting biological material, including blood tests and antibody testing, steroid therapy was administered in the form of an intravenous infusion of methylprednisolone at a total dose of 5.0 grams, followed by prednisone 60 mg once daily for 14 days with a target dose reduction of 5 mg every 5 days. In addition, antiepileptic treatment with levetiracetam was prescribed at a dose of 500 mg twice a day. In response to the identified ovarian mass, the patient was consulted with a gynecologist, and then, in accordance with the planned treatment, underwent laparascopic removal of the teratoma. Histopathological analysis confirmed the presence of a mature teratoma. After surgery, corticosteroids and intravenous immunoglobulins (IVIG) were continued to treat opsoclonus-myoclonus syndrome. After the therapy, a significant improvement in the patient’s condition was observed. The symptoms of opsoclonus and myoclonus were alleviated, cerebellar disorders were significantly reduced, and the patient’s fitness and clinical condition improved. This case highlights the importance of early diagnosis and intervention in opsoclonus-myoclonus syndrome, especially when it occurs in the context of a malignancy such as ovarian teratoma. Prompt diagnosis and treatment can lead to favorable clinical outcomes and significant improvements in patients’ quality of life.

The patient described underwent surgical removal of the tumor following their diagnosis. In the days after the surgery, we observed a gradual clinical improvement and resolution of the OMS symptoms. Currently, the patient remains under continuous medical care and is asymptomatic.

## Discussion

Opsoclonus-myoclonus syndrome (OMS) is a rare but complex neurological condition that can occur in both children and adults. This manuscript analyzes a variety of etiologies of OMS, including cancer, infective, and autoimmune causes. The results of the study support the literature that OMS often accompanies cancers such as ovarian teratoma, which has been extensively reported in the study by Battaller et al., where links between OMS and cancer have been identified, and by Miraclin et al., who have highlighted the complexity of cases involving teraatoms and NMDAR antibodies (7, 22). As in the work of Kinsbourne and Pohl et al., our study reveals that OMS, although less common in adults, tends to occur late in life, which often leads to delays in diagnosis and treatment (3, 5). Kranick et al. emphasize that the symptoms of OMS can be varied and ambiguous, which can cause difficulties in diagnosis and lead to misdiagnoses (26). It is also worth noting that the analysis of antibodies directed against neuronal surface antigens provides valuable information on the pathogenesis of OMS. The results of our observations are consistent with the findings of Sabater et al. and Armangué et al., who showed that the presence of these antibodies may be associated with immune-determined cases of OMS. Faster identification of these markers could lead to better diagnosis and more effective treatment of patients (13, 14).

Table 2 presents additional cases of OMS reported in the literature. The comparative characteristics included gender, age at diagnosis, methods of diagnosis, presence and types of identified antibodies, predominant symptoms, clinical diagnoses leading to OMS and treatments used.

**Table 2:** A selection of documented cases of Ospoclone-myoclonus syndrome found in the literature, along with their specific characteristics.

Sex	Age	Symptoms	Diagnostic tests	Identified antibodies	Diagnosis causing OMS	Treatment methods	Reference
Male	41 years	- Headaches in the occipital region - Sensitivity to light (photophobia) - Nausea and vomiting - Fever - Muscle pain - Difficulties with gait and balance - Nystagmus (involuntary eye movement) when looking to the right, followed by multidirectional nystagmus - Myoclonus (muscle jerks) - Dysarthric speech (slurred speech) - Hearing loss	- Examination of cerebrospinal fluid - CT scan of the abdomen - MRI of the head - EEG - Laboratory tests - Immunological and serological tests for: HIV, syphilis, HSV, VZV, EBV	In the IgM class in the direction of *Borrelia burgdorferi* infection.	Lyme disease	- Vaplroic acid - Acyclovir - Ampicillin - Methyloprednisolone - Intravenous immunoglobulin - Ceftriaxone - Doxycyline	(28)
Female	46 years	- Malaise - Headaches and dizziness - Muscle aches and pains - Double vision - Speech disorders - Behavioral and emotional disturbances	- Laboratory tests - Head CT scan - MRI scan of the head - Cerebrospinal fluid examination - EEG	Anti-MNDA	Teratoma	- Methyloprednisolone - Prednisone - Diazepam - Phenytoin - Valproic acid - Leveriacetam - Plasmapheresis	(29)
Female	18-months	- Gait disturbances - Swallowing disorders - Speech disorders - Opsoclonus - Myoclonus affecting the head, trunk, and limbs.	- MRI examination of the head and cervical spine - Cerebrospinal fluid analysis - Abdominal ultrasound - Chest X-ray - EEG - Tests for bacterial and viral infections - Abdominal CT scan	Not found	Neuroblastoma located in the left adrenal gland	Surgical removal of the tumor	(30)
Female	29 years	- Opsoclonus - Behavioral disorders manifesting as increased timidity and aggression - Myoclonus - Trunk and limb ataxia - Dysdiadochokinesis with asymmetry and a wide-based ataxic gait - Hallucinations	- MRI of the head - Cerebrospinal fluid examination - Whole body PET scan - Ultrasound of pelvic organs	Antibodies to the NMDA receptor	Autoimmune encephalitis	- Methylprednisolone - Antipsychotics - Benzodiazepines - Rituximab - Bortezomib	(22)
Female	28 years	- Gait ataxia - Involuntary movements - Double vision - Convulsions - Anhedonia - Functional myoclonus - Dystonia with rigidity in the upper and lower extremities - Dysdiadochokinesis - Impaired gait - Hyperreflexia with a pendulum knee reflex - Wide-based, ataxic gait	- MRI of the head - Examination of cerebrospinal fluid - Whole-body PET scan	Not found	Teratoma	- Therapeutic plasma exchange - Methylprednisolone - Surgical removal of the tumor - Rituximab	(22)
Female	14 years	- Discomfort in the cervical spine - Disorders of concentration and attention - Jerky-type movements - Difficulty walking - Objects falling from the hands - Nystagmus - Myoclonus - Gait ataxia	- MRI of the head - Cerebrospinal fluid examination - Tests for autoimmune antibodies - EEG	Not found	Teratoma	- Clonazepam - Immunoglobulins (IVIG) - Methylprednisolone - Surgical removal of tumor - Bleomycin, etoposide, and cisplatin	(31)

CT, Computer tomography; MRI, Magnetic resonance image; EEG, Electroencephalography; HIV, Human immunodeficiency virus; HSV, hepres simplex virus; VZV, varicella zoster virus; EBV, Epstein–Barr virus; PET, positron emission tomography; IVIG, immunoglobulin G.

## Conclusion

Although opsoclonus-myoclonus syndrome is a rare disease, it requires appropriate and applied treatment aimed at the etiology. In idiopathic and paraneoplastic cases, treatment and its results are relatively difficult to evaluate and compare. This is due to the high variability of the drugs used, as well as the number of patients. Currently, commonly used therapeutic options include: corticosteroid therapy, intravenous immunoglobulin administration and plasmapheresis (11). Patients with parainfectious OMS have a favorable prognosis and achieve full recovery (20, 27). In the case of patients in whom the etiological factor of the syndrome is tumors, treatment is based on resection of the lesion, which contributes to the improvement of the patient’s clinical condition. Our report highlights the need for prospective studies to better understand the pathogenesis of OMS and to identify potential biomarkers. Understanding the molecular mechanisms underlying OMS could lead to the development of new therapeutic strategies. Proper management of patients with OMS requires collaboration between neurologists, oncologists, surgeons, and immunologists. A multidisciplinary approach is essential to optimize the diagnosis, treatment and monitoring of patients with this rare condition. Increasing awareness of OMS among professionals is key to enabling faster and accurate diagnosis. Education and training materials can help identify the symptoms of OMS and refer patients to appropriate specialists. Patients with OMS should undergo long-term monitoring to assess neurological complications and the effectiveness of therapy. Regular check-ups and psychological support can significantly improve the quality of life of these patients.

## Data Availability

The raw data supporting the conclusions of this article will be made available by the authors, without undue reservation.
